# Data on melanin production in B16F1 melanoma cells in the presence of emu oil

**DOI:** 10.1016/j.dib.2016.11.039

**Published:** 2016-11-17

**Authors:** Minoru Ito, Kazuhiro Minami, Yoshimasa Sagane, Toshihiro Watanabe, Koichi Niwa

**Affiliations:** a Groupwide Research and Development, Noevir Co. Ltd., 112-1, Okadacho, Higashiohmi-shi, Shiga 527-0057, Japan; b Department of Food and Cosmetic Science, Faculty of Bioindustry, Tokyo University of Agriculture, 196 Yasaka, Abashiri 099-2493, Japan

**Keywords:** Emu oil, Melanoma, Melanogenesis, *Dromaius novaehollandiae*

## Abstract

Here, we present data on the effects of emu oil, obtained from emu (*Dromaius novaehollandiae*) fat deposits, on melanogenesis in B16F1 murine melanoma cells. The cells were cultured in media containing different concentrations of emu oil, and the melanin content of these cells was measured using a microplate reader. Next, melanin content was measured for cells cultured with α-melanocyte-stimulating hormone. This article reports the different melanin contents as μg melanin/mg cellular protein, by using bar graphs with error bars. The present data imply that emu oil reduces the cellular melanin production.

**Specifications Table**

**Table:** 

Subject area	*Biology*
More specific subject area	*Cell biology*
Type of data	*Figures*
How data was acquired	*Measurement of melanin content in the cell, using a microplate reader (MPR-A4i; Tosoh, Tokyo, Japan)*
Data format	*Analyzed*
Experimental factors	*Commercially available emu oil was purchased from Tokyo Nodai Bioindustry Co. Ltd., Japan*
Experimental features	*Melanin content was measured in B16F1 murine melanoma cells cultured in medium containing emu oil.*
Data source location	*Abashiri, Japan*
Data accessibility	*Data are presented in this article*

**Value of the data**

• The data presented indicate that emu oil reduces melanin production in B16F1 murine melanoma cells, but does not induce significant alternation in melanin production in α-MSH-stimulated cells.
• This is the first evidence for reduction of melanin production in cultured cells by emu oil.
• The data in this article provides useful knowledge for the cosmeceutical application of emu oil [1].

## 1 Data

**Fig. 1:**
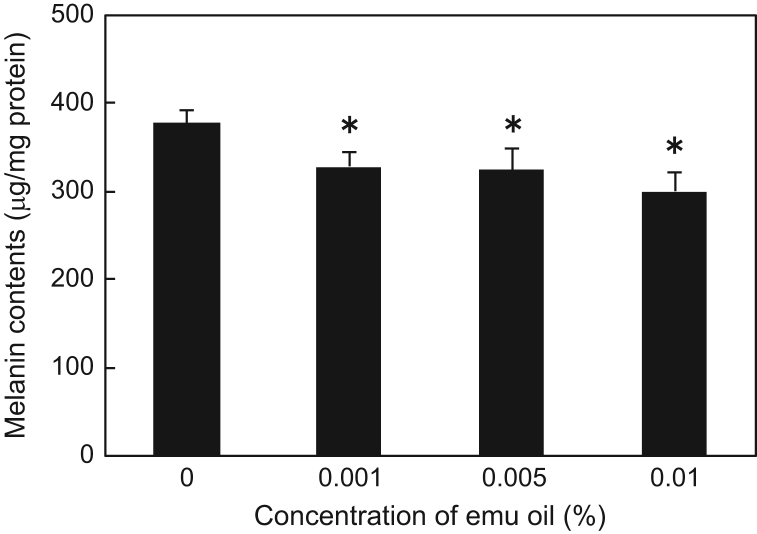
Melanin contents in B16F1 murine melanoma cells cultured in media containing various concentrations of emu oil. Data represent the mean±SD, *n*=3–4. ^⁎^, *P*<0.01 compared to 0% emu oil (non-repeated ANOVA followed by Bonferroni correction).

**Fig. 2:**
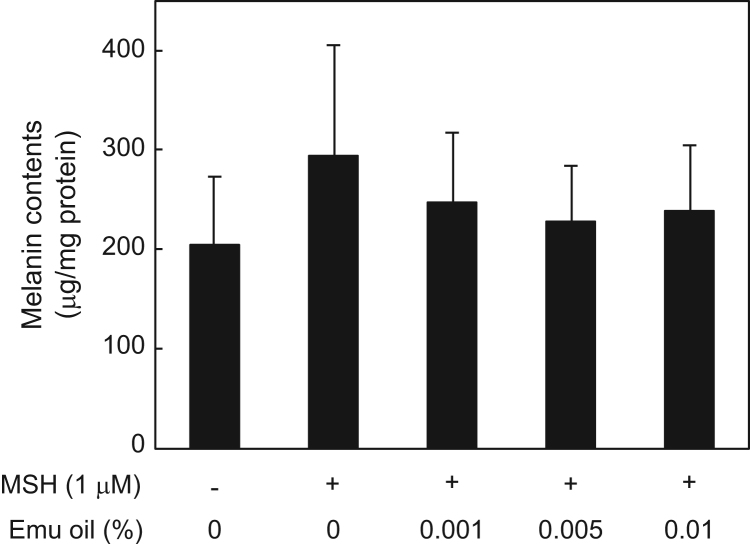
Melanin contents in B16F1 murine melanoma cells cultured in media containing the α-melanocyte-stimulating hormone (α-MSH) and various concentrations of emu oil. Data represent the mean±SD, *n*=3.

Here, we present data on the melanin contents in B16F1 murine melanoma cells per total protein content in the cells, in culture medium containing 0, 0.001, 0.005, and 0.01% emu oil (Fig. 1). The data show significant reduction of melanin production in the presence of emu oil. However, Fig. 2 indicates that the melanin content in the presence of 1 µM α-melanocyte-stimulating hormone (α-MSH) was not significantly altered despite supplementation with emu oil.

## 2 Experimental design

Melanin, which is the major pigment of skin, plays an important role in protection against UV light under normal physiological conditions. However, overproduction of the melanin causes cosmetic problems, such as staining and freckles on the skin. Here, we examined the melanin production in murine B16F1 melanoma cells in the presence of emu oil, which is widely utilized in cosmetics for its moisturizing and transdermal penetration enhancing properties [1]. In this study, we measured the melanin contents in B16F1 cells treated with various concentrations of emu oil. The melanin contents were measured in the cells in both the presence and absence of the α-MSH, which is one of the endogenous factors that regulate melanogenesis. The measured melanin contents were divided by the cellular protein amount in each sample to compare the cellular melanin production.

## 3 Materials and methods

### 3.1 Materials

Commercially available emu oil was purchased from Tokyo Nodai Bioindustry Co. Ltd. (Abashiri, Japan). B16F1 murine melanoma cells were obtained from Riken BioResource Center (Tsukuba, Japan).

### 3.2 Cell culture and emu oil treatment

B16F1 cells were maintained in a CO_2_ incubator in Dulbecco׳s modified Eagle medium (DMEM) supplemented with 10% fetal bovine serum, penicillin (100 U/ml) and streptomycin (100 μg/ml). To disperse the hydrophobic emu oil into the culture medium, fatty-acid-free bovine serum albumin (BSA) was added to the culture medium as an emulsifier [2], [3]. Thus, emu oil was added to culture medium containing 4% fatty-acid-free BSA (DMEM-BSA) at concentrations of 0.001, 0.005, and 0.01%. B16F1 cells were incubated in CO_2_ for 24 h after being seeded in 10-cm diameter culture dishes containing DMEM at a density of 1×10^4^ cells/cm^2^. The culture media were then replaced with 8 ml of DMEM-BSA with or without emu oil. The cells were further incubated for 72 h and used for measurement of melanin content.

### 3.3 α-MSH treatment

The culture medium containing 1 μM α-MSH was prepared by adding the stock solution of α-MSH (100 μM in distilled water) to the medium at a ratio of 1:100. B16F1 cells were incubated in a CO_2_ incubator for 24 h after being seeded in 10-cm diameter culture dishes containing DMEM at a density of 1×10 ^4^ cells/cm^2^. The culture medium was then replaced with 8 ml of medium containing DMEM-BSA, DMEM-BSA with 1 μM α-MSH or DMEM-BSA with 1 μM α-MSH and emu oil. The cells were further incubated for 72 h and used for measurement of melanin content.

### 3.4 Measurement of melanin content in the melanoma cells

After incubation with emu oil or emu oil and α-MSH, the cells were harvested and washed with phosphate-buffered saline thrice by centrifugation (1500 rpm, 5 min). The melanin contents in the resultant cell pellets were determined according to previously described methods with a slight modification [4], [5], [6]. The cell pellets were dissolved in 500 μl of 0.5 N NaOH and incubated at 60 °C for 1 h. The absorbance of each sample at 450 nm was measured using a microplate reader (MPR-A4i; Tosoh, Tokyo, Japan), and converted to melanin concentration using a standard curve generated with commercially available melanin (Sigma-Aldrich, St. Louis, MO, USA). The protein concentration of each sample was determined using a protein assay kit (Pierce, IL, USA). The measured melanin contents were then divided by the protein amount and expressed as the μg/mg of cell protein.

### 3.5 Statistical analysis

The values were expressed as the mean±standard deviation. The differences in mean values were assessed with non-repeated ANOVA followed by Bonferroni multiple comparisons. Significance was considered when is *p*<0.05.
